# Hematologic and plasma biochemical prognostic indicators for stranded free-ranging phocids presented for rehabilitation

**DOI:** 10.1038/s41598-022-14923-2

**Published:** 2022-06-22

**Authors:** Matthew Vail, Hugues Beaufrère, Stefan Gallini, Hubert Paluch, João Brandão, Peter M. DiGeronimo

**Affiliations:** 1 Department of Clinical Sciences and Advanced Medicine, School of Veterinary Medicine, University of Pennsylvania, 3900 Delancey Street, Philadelphia, PA 19104 USA; 2 Department of Clinical Studies, Ontario Veterinary College, University of Guelph, 50 Stone Road E., Guelph, ON N1G 2W1 Canada; 3 Marine Mammal Stranding Center, 3625 Atlantic Brigantine Blvd., Brigantine, NJ 08203 USA; 4 Department of Veterinary Clinical Sciences, College of Veterinary Medicine, Oklahoma State University, 2065 W. Farm Rd., Stillwater, OK 74078 USA; 5 Adventure Aquarium, 1 Riverside Drive, Camden, NJ 08103 USA; 6 Present Address: Philadelphia Zoo, 3400 W. Girard Avenue, Philadelphia, PA 19104 USA

**Keywords:** Animal physiology, Risk factors

## Abstract

This retrospective study used data obtained from medical records of 530 stranded free-ranging harbor (*Phoca vitulina*), grey (*Halichoerus grypus*), harp (*Pagophilus groenlandicus*), and hooded seals (*Cystophora cristata*) presented to the Marine Mammal Stranding Center in Brigantine, New Jersey from January 1998 through December 2016. The objective was to identify hematological and plasma biochemical parameters of seals at time of presentation that were associated with successful rehabilitation and with duration of hospitalization using univariate and multivariate logistic regressions. At presentation, animals that subsequently survived rehabilitation had greater alkaline phosphatase activity and absolute lymphocyte and total calcium concentrations and lower blood urea nitrogen, sodium, chloride, phosphorus, and total bilirubin concentrations and lower aspartate aminotransferase and alanine aminotransferase activities than animals that eventually died or were euthanized while under care. Results suggest that young, actively growing animals are more likely to survive rehabilitation and that bloodwork consistent with dehydration, systemic disease, and exhaustion are negative prognostic indicators. These results provide prognostic indicators that may aid clinical decision-making for seals presented for rehabilitation.

## Introduction

Across the globe, biodiversity is increasingly threatened by a variety of anthropogenic factors. Marine mammals include pinnipeds, cetaceans, sirenians, sea otters (*Enhydra lutris*) and the polar bear (*Ursus maritimus*) and have been classified as disproportionally threatened relative to other groups of mammals^1^. Most species of marine mammals are directly threatened by human activities, such as commercial fisheries, direct harvesting, urban development, traffic and tourism, and more than half of core marine mammal habitats are considered at risk^2^. Of all families of marine mammals, Phocidae face the second largest number of threats and the Atlantic coast of the United States is considered a hotspot of risks to phocid populations^2^.

Several seal species inhabit the coastal waters of the northeastern United States over the course of the year, including harbor (*Phoca vitulina*), grey (*Halichoerus grypus*), harp (*Pagophilus groenlandicus*), and hooded seals (*Cystophora cristata*). These North Atlantic phocid populations represent a significant proportion of global seal populations and play an important role in structuring the North Atlantic ecosystem^3^. Additionally, some of these species are harvested for commercial and subsistence purpose^3^. Therefore, their preservation is essential.

Due to close proximity with human populations, individual seals are commonly found stranded on land, unable or unwilling to return to water even when approached by people. Individuals may become systemically debilitated and strand along the coast often due to illness, injury, maternal abandonment, and negative human interaction^4^. Few wildlife rehabilitation centers are appropriately equipped and meet federal regulations to accommodate marine mammals. Those that do can be faced with heavy caseloads that originate across a wide geographic distribution. This, along with the costs of maintaining marine mammals under human care, make phocid rehabilitation labor-intensive and expensive. Accurate triage and identification of prognostic indicators can help to allocate resources to those patients most likely to survive and to limit animal suffering by recognizing indications for humane euthanasia.

Studies that determine prognostic indicators in wildlife rehabilitation are increasingly popular, however these studies are specific to the species, populations, and clinics in which they were conducted and can be challenging to broadly apply due to differences in standard operating procedures between rehabilitation centers. In human medicine, prognostic indicators have been defined as “any variable that is associated with the risk of a subsequent health outcome among people with a particular health condition”^5^. In wildlife medicine, due to the wide range of presentations and challenges in diagnosing specific diseases, prognostic indicators are typically broad. Nevertheless, these allow wildlife veterinarians and/or rehabilitators to gauge the chances of survival and/or release back into the wild for a given individual. Furthermore, identification of prognostic indicators in wildlife rehabilitation can improve animal welfare, optimize the use of funds and resources, and minimize the spread of infectious diseases. Previous research has demonstrated that blood values indicate clinical and physiologic changes in phocids undergoing rehabilitation and provide an objective measure of when a seal is eligible for release back into the wild^4,6,7^.

This study investigated if blood analytes at the time of presentation can serve as prognostic indicators for successful release. The goal of this research was to identify hematologic and plasma biochemical values of stranded phocids at time of presentation with the probability of successful release, and to evaluate the association of these values with length of hospitalization.

## Materials and methods

### Case definition

Medical records from all free-ranging phocids admitted to the Marine Mammal Stranding Center (MMSC) in Brigantine, New Jersey from January 1998 through December 2016 were available for review. Inclusion criteria were all harbor, grey, harp, and hooded seals for which age class, sex, term of hospitalization, and final disposition were recorded and for which hematology and/or blood biochemistry results were obtained within 48 h of admission. Cases with missing data were excluded from the analysis. Sex was categorized as male, female, or undetermined, and age class as neonatal, juvenile, or adult based on external anatomical and morphological characteristics including body length and mass, pelage, tooth development and umbilicus^6^. Animals that had lanugo and an umbilical stump present were categorized as neonates, whereas juveniles had no umbilical stump, may or may not have some lanugo present, and were smaller than sexually mature adults of their species. Term of hospitalization was determined as number of days from presentation to final disposition. Final disposition included those animals that were successfully released to natural habitat and those that were not released. Animals not released included those that died naturally while under human care, those that were euthanized, and those that were deemed unsuitable for release and transferred to another facility. Animals were deemed unsuitable for release if they had permanent physical impairment that may have limited their ability to capture prey, migrate, or reproduce or if they had a chronic medical condition that required ongoing treatment and if they could experience appropriate welfare under managed care. These cases were determined at the discretion of the attending veterinarian in accordance with local and federal law. Data was recorded using a spreadsheet and software package (Microsoft Excel, Microsoft Corporation, Redmond, WA 98052, USA).

### Hematologic and plasma biochemistry profiles

Only cases which included a hematologic or biochemistry profile dated within 48 h of the date of admission were included. For cases for which more than one hematologic or biochemistry profile were obtained, only those from the date closest to admission were included. In all cases, hematology was performed on whole blood in potassium EDTA blood collection tubes and biochemistry performed on lithium heparinized plasma at a commercial veterinary diagnostic laboratory (Antech Diagnostics, Fountain Valley, CA 92708, USA). Hematology parameters evaluated included hematocrit (HCT %) and concentrations of hemoglobin (g/dL), leukocytes (WBC/µL), erythrocytes (RBC × 10^3^/µL), platelets (× 10^3^/µL), and relative and absolute neutrophils (/µL), band neutrophils (/µL), lymphocytes (/µL), monocytes (/µL), eosinophils (/µL), and basophils (/µL). Plasma biochemistry analytes evaluated included measured concentrations of total protein (TP g/dL), albumin (g/dL), total bilirubin (U/L), blood urea nitrogen (BUN mg/dL), creatinine (mg/dL), phosphorus (P mg/dL), glucose (mg/dL), total calcium (tCa^2+^ mg/dL), sodium (Na^+^ mEq/L), potassium (K^+^ mEq/L), chloride (Cl^−^ mEq/L), cholesterol (mg/dL), activities of aspartate aminotransferase (AST U/L), alanine aminotransferase (ALT U/L), alkaline phosphatase (ALP U/L), creatine kinase (U/L), and amylase (U/L), and calculated globulin concentrations (g/dL), albumin:globulin, BUN:creatinine, and Na^+^:K^+^ ratios. Not every analyte was available for each individual.

### Descriptive statistical analyses

Normality was assessed by visual evaluation of distribution and assessment of kurtosis and skewness of the data. The means of each analyte were compared among seals that were successfully released, those that were transferred, those euthanized, and those that died under care by one-way analysis of variance (ANOVA) (hemoglobin, HCT, relative neutrophils, relative lymphocytes, absolute and relative monocytes, TP, albumin, globulin, Na^+^, cholesterol, and amylase) or Kruskal–Wallis test (length of hospitalization, WBC, RBC, platelets, absolute neutrophils, absolute and relative band neutrophils, absolute lymphocytes, absolute and relative eosinophils, absolute and relative basophils, AST, ALT, ALP, total bilirubin, BUN, creatinine, tCa^2+^, P, Cl^−^, K^+^, glucose and CPK) depending on whether the data were normally distributed or not, respectively. Statistical significance was defined as *P* < 0.05 and analysis was performed using a commercially available statistical software (JASP, Version 0.14, 2020, Amsterdam, The Netherlands).

### Statistical analyses for predictors of release and length of hospitalization

The probability of successful release was modelled using univariate and multiple logistic regressions. Parameters that were significant in the univariate models after applying a false discovery rate of 5% were incorporated into the multiple logistic model. These were retained following a backward selection process with the final model only containing significant predictors. Correlation and multicollinearity among continuous predictors were assessed using a Pearson correlation coefficient matrix and variance inflation factors (VIF). Only the most significant of the highly correlated parameters were retained in the final multiple logistic model based on the lowest Akaike information criterion (AIC). Parameter estimates were exponentiated to obtain odds ratios. The fit of the final model was assessed using the area under the receiver operating characteristic (ROC) curve. Other assumptions were checked on residual plots. Seal species were included in the model as dummy variables.

The number of days in hospitalization was modelled using univariate and multiple negative binomial regression models. Only data from seals successfully rehabilitated were included in these models. Parameters that were significant on the univariate models were incorporated and retained into the multiple negative binomial model as described above. Parameter estimates were exponentiated to obtain multiplicative factors on expected counts (number of days in hospital). Assumptions and fit were checked on residual plots. Univariate and multivariate log-linear models were also performed, but overdispersion was an issue in most models.

An alpha of 0.05 was used for statistical significance and analysis was performed with the software R (R foundation for statistical computing, 2019, Vienna, Austria).

## Results

### Descriptive statistics

**Figure 1:**
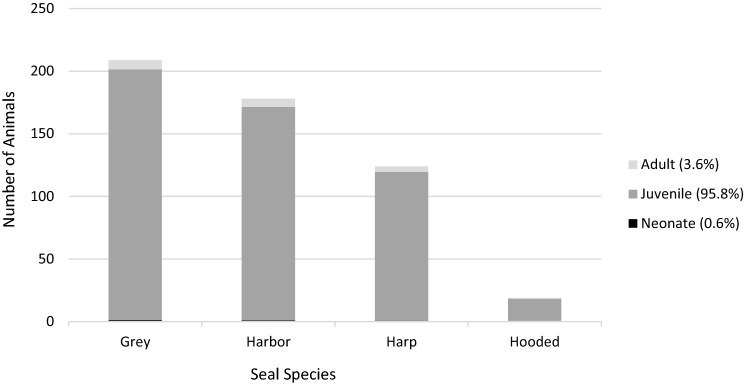
Number and age distribution of free-ranging harbor (*Phoca vitulina*), grey (*Halichoerus grypus*), harp (*Pagophilus groenlandicus*), and hooded (*Cystophora cristata*) seals presented to New Jersey Marine Mammal Stranding Center from January 1998 through December 2016.

During the study period, a total of 3,148 animals were presented to the MMSC. Of these, 1,159 were phocids. One phocid was excluded from analysis because final disposition was not documented and an additional 24 phocids were excluded because species was not documented in the medical records. Of the remaining 1,134 harbor, grey, harp, and hooded seals, hematology (*n* = 526) and/or plasma biochemistry (*n* = 529) results were obtained within 48 h of presentation from 530 (46.7%) animals. The sample population was comprised of 39.4% grey (*n* = 209), 33.6% harbor (*n* = 178), 23.4% harp (*n* = 124), and 3.6% hooded seals (*n* = 19). Of these, 56.2% were male (*n* = 298), 43.2% female (*n* = 229) and 0.6% of unknown sex (*n* = 3), and 3.6% were adults (*n* = 3 grey, 1 harbor, and 15 harp seals), 95.8% juveniles (*n* = 205 grey, 175 harbor, 109 harp, and 19 hooded seals), and 0.6% neonates (*n* = 1 grey and 2 harbor seals) (Fig. 1). Of the 530 animals, 77.2% were successfully released (*n* = 409). Of those not released, 57% died under care (*n* = 69), 24.0% were euthanized (*n* = 29), and 18.2% were transferred to another facility (*n* = 22).

Time spent hospitalized differed significantly between groups (*P* < 0.001). Animals that were released spent a median of 41 days hospitalized (range 0–278; mean: 47.2 ± 18.9) whereas those transferred to another facility to be kept under long-term managed care spent a median of 26.5 days hospitalized (range 0–230; mean: 61.8 ± 72.8). Animals were euthanized at a median of 3 days following presentation (range 0–99; mean: 15.5 ± 26.9) whereas those that died did so at a median of 5.5 days after presentation (range 0–152; mean: 9.1 ± 18.9).

Hematologic and plasma biochemical profiles for seals that were successfully released and those that were not are summarized in Tables 1 and 2, respectively. Several analytes differed significantly among groups with AST, ALT, bilirubin, BUN, P, Na^+^ and Cl^−^ being lower, and ALP, tCa^2+^ and absolute lymphocytes being higher in seals that were successfully released and those that survived rehabilitation to be transferred to another facility versus those that died or were euthanized while under care. Absolute and relative eosinophils were highest and red blood cells lowest in seals that were euthanized while under care.

**Table 1:** Summary of descriptive statistics for hematology profiles from free ranging seals that survived rehabilitation and were successfully released or transferred to another institution and those that were euthanized or died while under care.

Analyte	Released			Transferred			Euthanized			Died			p-value
	N	Mean ± SD	Median (range)	N	Mean ± SD	Median (range)	N	Mean ± SD	Median (range)	N	Mean ± SD	Median (range)	
Hemoglobin* (g/dL)	407	17.3 ± 4.0	17.0 (1.3–30.1)	22	16.6 ± 2.7	17.0 (9.4–21.2)	29	16.4 ± 3.6	16.4 (9.5–24.6)	68	17.9 ± 5.0	16.6 (5.1–28.6)	0.368
HCT* %	406	48 ± 11	48 (5–80)	22	48 ± 8	50 (28–57)	29	47 ± 10	47 (29–65)	68	51 ± 14	48 (15–86)	0.325
RBC (× 10^6^/µL)	**405**	**4.47 ± 4.28**	**4.31 (1.50–89.00)**	**22**	**4.33 ± 0.71**	**4.55 (2.70–5.30)**	**29**	**3.96 ± 0.69**	**13.90 (4.40–82.90)**	**68**	**4.56 ± 1.04**	**4.41 (1.41–7.20)**	**0.011**
Platelets (× 10^3^/µL)	389	6236 ± 78,731	581 (32–1100 × 10^3^)	19	667 ± 227	620 (270–1365)	24	509 ± 248	473 (144–992)	64	16,175 ± 124,930	16,175 (25–1000 × 10^3^)	0.183
WBC (× 10^3^/µL)	406	15.40 ± 7.18	14.30 (1.90–65.00)	22	14.92 ± 7.20	13.10 (6.90–31.90)	29	18.30 ± 15.43	13.90 (4.40–82.90)	68	13.39 ± 6.55	12.7 (2.50–43.70)	0.127
Neutrophils/µL	404	11,501 ± 6311	10,110 (1444–52,650)	22	10,969 ± 6416	9430 (3960–29,348)	29	12,545 ± 8338	9690 (1848–42,930)	68	10,115 ± 5733	9038 (1400–39,767)	0.336
% Neutrophils*	404	72.8 ± 9.3	74.0 (42.0–93.0)	22	71.2 ± 11.2	73.0 (40.0–92.0)	29	75.2 ± 16.5	80.0 (8.0–92.0)	68	73.8 ± 8.5	76.0 (53.0–91.0)	0.269
Band Neutrophils/µL	404	29 ± 173	0 (0–2396)	22	53 ± 171	0 (0–638)	29	72 ± 260	0 (0–1316)	68	79 ± 314	0 (0–1890)	0.275
% Band Neutrophils	404	0.1 ± 0.6	0 (0–4.0)	22	0.3 ± 1.1	0 (0–5.0)	29	0.3 ± 0.9	0 (0–4.0)	68	0.5 ± 2.4	0 (0–18.0)	0.261
Lymphocytes/µL	**404**	**2368 ± 1217**	**2247 (195–9842)**	**22**	**2432 ± 1069**	**2534 (828–4512)**	**29**	**2016 ± 1118**	**1771 (486–4720)**	**68**	**1954 ± 992**	**1967 (157–4584)**	**0.019**
% Lymphocytes*	404	16.7 ± 7.8	16.0 (1.0–53.0)	22	18.0 ± 9.0	16.0 (7.0–44.0)	29	14.1 ± 8.5	14.0 (2.0–43.0)	68	16.1 ± 8.0	15.0 (1.0–38.0)	0.269
Monocytes/µL*	404	1073 ± 631	954 (0–4336)	22	1069 ± 622	960 (0–2460)	29	1023 ± 845	702 (118–4145)	67	891 ± 545	770 (35–2329)	0.189
% Monocytes*	404	7.1 ± 3.3	7.0 (0–25.0)	22	7.3 ± 3.1	8.0 (0–14.0)	29	5.9 ± 2.6	6.0 (1.0–11.0)	67	6.8 ± 2.9	7.0 (1.0–17.0)	0.163
Eosinophils/µL	**404**	**377 ± 377**	**269 (0–2964)**	**22**	**407 ± 293**	**314 (0–1148)**	**29**	**2543 ± 12,746**	**117 (0–68,807)**	**67**	**282 ± 262**	**174 (0–1056)**	**0.004**
% Eosinophils	**404**	**2.7 ± 2.8**	**2.0 (0–19.0)**	**22**	**2.9 ± 2.2**	**2.5 (0–9.0)**	**29**	**4.3 ± 15.3**	**1.0 (0–83.0)**	**67**	**2.3 ± 2.1**	**2.0 (0–11.0)**	**0.020**
Basophils/µL	404	77 ± 132	0 (0–858)	22	129 ± 207	98 (0–957)	29	104 ± 313	0 (0–1658)	67	69 ± 139	0 (0–768)	0.137
% Basophils	404	0.5 ± 0.9	0 (0–7.0)	22	0.8 ± 0.8	1.0 (0–3.0)	29	0.3 ± 0.6	0 (0–2.0)	67	0.5 ± 1.0	0 (0–5.0)	0.112

Sample (N), mean and standard deviation (SD), median and range are provided for each analyte. Analytes were analyzed by ANOVA (indicated by *) or Kruskal–Wallis test depending on the normality of their distribution and those that are statistically significantly different among groups (p-value < 0.05) are indicated in bold.

**Table 2:** Summary of descriptive statistics for plasma biochemistry profiles from free-ranging seals that survived rehabilitation and were successfully released or transferred to another institution and those that were euthanized or died while under care.

Analyte	Released			Transferred			Euthanized				Died			p-value
	N	Mean ± SD	Median (range)	N	Mean ± SD	Median (range)	N		Mean ± SD	Median (range)	N	Mean ± SD	Median (range)	
Total protein* (g/dL)	409	7.3 ± 1.2	7.3 (4.6–11.2)	22	7.0 ± 1.4	6.9 (4.9–11.6)	28		7.3 ± 1.6	7.1 (4.6–10.8)	69	7.1 ± 1.4	7.0 (4.4–10.2)	0.624
Albumin* (g/dL)	409	2.8 ± 0.5	2.8 (1.4–4.6)	22	2.9 ± 0.5	2.8 (2.0–3.9)	28		2.7 ± 0.6	2.7 (1.4–3.7)	69	2.7 ± 0.5	2.6 (1.8–4.5)	0.097
Globulin* (g/dL)	407	4.5 ± 1.2	4.4 (1.4–8.7)	22	4.1 ± 1.5	4.0 (1.8–9.1)	28		4.6 ± 1.5	4.2 (2.1–7.7)	69	4.5 ± 1.3	4.4 (1.5–8.0)	0.575
AST (U/L)	**409**	**167 ± 249**	**113 (32–3630)**	**21**	**92 ± 50**	**83 (29–283)**	**28**		**213 ± 972**	**120 (37–1113)**	**69**	**255 ± 332**	**142 (45–2188)**	** < 0.001**
ALT (U/L)	**409**	**64 ± 63**	**48 (9–690)**	**22**	**43 ± 22**	**39 (12–114)**	**28**		**69 ± 85**	**35 (6–320)**	**69**	**83 ± 90**	**53 (14–533)**	**0.025**
ALP (U/L)	**403**	**57 ± 51**	**44 (0–388)**	**22**	**100 ± 93**	**71 (17–441)**	**28**		**43 ± 26**	**39 (1–99)**	**68**	**49 ± 35**	**44 (4–203)**	** < 0.001**
Total bilirubin (mg/dL)	**409**	**0.3 ± 0.2**	**0.2 (0.1–1.1)**	**22**	**0.3 ± 0.2**	**0.3 (0.1–0.8)**	**28**		**0.3 ± 0.2**	**0.3 (0.1–0.9)**	**69**	**0.4 ± 0.3**	**0.3 (0.1–1.4)**	** < 0.001**
BUN (mg/dL)	**409**	**48 ± 21**	**44 (5–233)**	**22**	**45 ± 10**	**43 (30–61)**	**28**		**64 ± 41**	**47 (10–165)**	**69**	**76 ± 56**	**52 (20–309)**	** < 0.001**
Creatinine (mg/dL)	409	0.5 ± 0.3	0.4 (0.1–3.8)	22	0.4 ± 0.2	0.3 (0.2–0.9)	28		0.8 ± 0.9	0.5 (0.2–4.5)	69	1.1 ± 1.6	0.4 (0.1–7.5)	0.090
Total Ca (mg/dL)	**409**	**8.8 ± 0.6**	**8.8 (6.5–10.6)**	**22**	**9.0 ± 0.8**	**9.3 (7.0–10.2)**	**28**		**8.5 ± 0.8**	**8.7 (7.0–9.8)**	**69**	**8.5 ± 0.9**	**8.3 (7.1–12.8)**	** < 0.001**
P (mg/dL)	**409**	**6.8 ± 1.3**	**6.6 (0.2–13.4)**	**22**	**6.9 ± 1.5**	**7.0 (3.2–9.5)**	**28**		**7.3 ± 3.1**	**6.7 (3.8–17.5)**	**69**	**8.5 ± 3.8**	**7.2 (4.0–24.6)**	**0.031**
Na^+^* (mEq/L)	**409**	**154 ± 9**	**152 (140–213)**	**22**	**149 ± 4**	**148 (140–156)**	**28**		**160 ± 17**	**156 (136–207)**	**69**	**162 ± 17**	**155 (142–209)**	** < 0.001**
Cl^-^ (mEq/L)	**408**	**111 ± 10**	**109 (96–208)**	**22**	**107 ± 4**	**107 (99–116)**	**28**		**117 ± 15**	**113 (89–159)**	**69**	**119 ± 16**	**113 (101–172)**	** < 0.001**
K^+^ (mEq/L)	409	4.4 ± 0.6	4.4 (3.1–8.2)	22	4.4 ± 0.5	4.5 (3.3–5.3)	28		4.4 ± 1.0	4.2 (2.5–7.0)	68	4.7 ± 1.0	4.4 (3.0–8.5)	0.308
Glucose (mg/dL)	409	154 ± 37	150 (22–362)	22	167 ± 34	158 (131–246)		28	159 ± 76	144 (34–500)	69	159 ± 72	159 (131–246)	0.285
Cholesterol* (mg/dL)	408	284 ± 88	259 (150–720)	22	316 ± 105	271 (187–562)	28		282 ± 73	257 (180–516)	69	279 ± 89	260 (117–527)	0.373
CPK (U/L)	403	1520 ± 2641	784 (21–31,730)	22	1020 ± 1222	697 (126–4827)	25		2130 ± 6857	508 (129–34,860)	69	2329 ± 4257	878 (84–24,655)	0.161
Amylase* (U/L)	403	540 ± 324	450 (1–2005)	22	345 ± 323	573 (138–1479)	27		639 ± 349	620 (6–1294)	68	499 ± 313	386 (4–1485)	0.122

Sample size (N), mean and standard deviation (SD), median and range are provided for each analyte. Analytes were analyzed by ANOVA (indicated by *) or Kruskal–Wallis test depending on the normality of their distribution and those that are statistically significantly different among groups (p-value < 0.05) are indicated in bold.

### Predictors of successful release

Results of univariate and multivariate logistic models are reported as odds ratios (OR) along with their 95% confidence intervals (CI). The univariate logistic model showed that the odds of successful release increased by 4% for each additional day in hospital (OR: 1.04; 95% CI 1.02–1.05), by 52% for each 1 mg/dL increase in tCa^2+^ (OR: 1.52; 95% CI 1.11–2.08). Conversely, the odds of successful release decreased by 89% for each 1 mg/dL increase in bilirubin (OR: 0.11; 95% CI: 0.04–0.34), by 17% for each 10 mg/dL increase in BUN (OR: 0.83; 95% CI 0.77–0.89), by 61% for each 1 mg/dL increase in creatinine (OR: 0.39; 95% CI 0.25–0.62), by 21% for each 1 mg/dL increase in P (OR: 0.79; 95% CI 0.71–0.87), by 28% for each 10 mEq/L increase in Na^+^ (OR: 0.72; 95% CI 0.62–0.85), and by 29% for each 10 mEq/L increase in Cl^−^ (OR: 0.71; 95% CI 0.60–0.84) (Table 3).

**Table 3:** Univariate significant variables for successful release of free-ranging seals (harbor [*Phoca vitulina*], grey [*Halichoerus grypus*], harp [*Pagophilus groenlandicus*], and hooded seals [*Cystophora cristata*]) presented for rehabilitation.

Variable	OR	95% CI	p-value
Days in hospital (/day)	1.04	1.02–1.05	< 0.001
Bilirubin (/1 mg/dL)	0.11	0.04–0.34	< 0.001
BUN (/10 mg/dL)	0.83	0.77–0.89	< 0.001
Creatinine (/1 mg/dL)	0.39	0.25–0.62	< 0.001
Phosphorus (/1 mg/dL)	0.79	0.71–0.87	< 0.001
Calcium (/1 mg/dL)	1.52	1.11–2.08	0.048
Sodium (/10 mmol/L)	0.72	0.62–0.85	< 0.001
Chloride (/10 mmol/L)	0.71	0.60–0.84	< 0.001

For the multivariate logistic models, highly correlated (e.g. BUN:creatinine, r = 0.71; and Na^+^:K^+^, r = 0.81) and non-significant variables were removed. Odds of successful release increased by 3% for each additional day of hospitalization (OR: 1.03; 95% CI 1.02–1.05) and by 51% for each 1 mg/dL increase in tCa^2+^ (OR: 1.51; 95% CI: 1.08–2.10), whereas the odds of release decreased by 2% for each 10 mg/dL increase in BUN (OR:0.98; 95% CI 0.97–0.99) (Table 4). There was a 3% increase in odds of successful release with each additional day in hospital when considering BUN and tCa^2+^ (CI 95, *P* < 0.001). The area under the curve (AUC) for the ROC curve for the final model was 0.82.

**Table 4:** Multivariate significant variables for successful release of free-ranging seals (harbor [*Phoca vitulina*], grey [*Halichoerus grypus*], harp [*Pagophilus groenlandicus*], and hooded seals [*Cystophora cristata*]) presented for rehabilitation.

Variable	OR	95% CI	p-value
Days in hospital (/day)	1.03	1.02–1.05	< 0.001
BUN (/10 mg/dL)	0.98	0.97–0.99	< 0.001
Calcium (/1 mg/dL)	1.51	1.08–2.10	0.01

### Predictors of term of hospitalization

In the univariate negative binomial models, grey seals and higher amylase concentrations were associated with increased number of days in hospital (Table 5). Number of days in hospital was 32% greater for grey seals compared to other seals (OR: 1.32; 95% CI 1.15–1.50). Length of hospitalization increased by 5% for each 100 mg/dL increase in amylase (OR: 1.05; 95% CI 1.03–1.07).

**Table 5:** Univariate significant predictors of an increased number of days in hospital of free-ranging seals (harbor [*Phoca vitulina*], grey [*Halichoerus grypus*], harp [*Pagophilus groenlandicus*], and hooded seals [*Cystophora cristata*]) presented for rehabilitation.

Variable	e^β^	95% CI	p-value
Grey seal	1.32	1.15–1.50	< 0.001
Harp seal	0.56	0.49–0.65	< 0.001
TP (/1 g/dL)	0.92	0.87–0.97	0.009
Albumin (/1 g/dL)	0.60	0.53–0.68	< 0.001
ALT (/100 U/L)	0.85	0.77–0.95	0.009
Creatinine (/1 mg/dL)	0.44	0.47–0.74	< 0.001
Phosphorus (/1 mg/dL)	0.91	0.87–0.96	0.002
Sodium (/10 mmol/L)	0.84	0.79–0.90	< 0.001
Potassium (/1 mmol/L)	0.85	0.75–0.96	0.028
Amylase (/100 U/dL)	1.05	1.03–1.07	< 0.001
Hematocrit (/10)	0.48	0.27–0.86	0.042
WBC (/1000/µL)	1.10	1.01–1.21	0.072
Eosinophils (/1000/µL)	0.80	0.67–0.95	0.042

The univariate negative binomial models also showed that harp seals and increased ALT activity and increased concentrations of TP, albumin, Na^+^, HCT, creatinine, P, K^+^, and eosinophils had statistically significant association with decreased number of days in hospital (Table 5). Harp seals were 44% less likely to have longer periods of hospitalization (OR: 0.56; 95% CI 0.49–0.65). The most influential of all factors decreasing hospital stay were creatinine concentration and HCT. Length of hospitalization decreased by 56% for each 1 mg/dL increase in creatinine (OR: 0.44; 95% CI 0.47–0.74) and by 52% for each 10% increase in HCT (OR: 0.48; 95% CI 0.27–0.86).

For the multivariate negative binomial model, after highly correlated and non-significant variables were removed, harp seals and increasing creatinine, Na^+^ and K^+^ concentrations were associated with shorter lengths of hospitalization (Table 6). Harp seals were least likely to spend more time hospitalized than other species (OR: 0.72; 95% CI 0.58–0.89). Length of hospitalization increased by 31% for each 1 mg/dL increase in creatinine (OR: 0.69; 95% CI 0.58–0.82), by 10% for each 10 mmol/L increase in Na^+^ (OR: 0.90; 95% CI 0.82–0.98), and by 15% for each mmol/L increase in K^+^ (OR: 0.85; 95% CI 0.78–0.93).

**Table 6:** Multivariate significant predictors of an increased number of days in hospital of free-ranging seals (harbor [*Phoca vitulina*], grey [*Halichoerus grypus*], harp [*Pagophilus groenlandicus*], and hooded seals [*Cystophora cristata*]) presented for rehabilitation.

Variable	e^β^	95% CI	p-value
Harp seal	0.72	0.58–0.89	0.002
Creatinine (/1 mg/dL)	0.69	0.58–0.82	< 0.001
Sodium (/10 mmol/L)	0.90	0.82–0.98	0.021
Potassium (/1 mmol/L)	0.85	0.78–0.93	< 0.001
WBC (/10)	1.13	1.02–1.24	0.017

## Discussion

### Hematologic and plasma biochemical parameters

Several hematologic and plasma biochemical analytes significantly differed between seals that successfully underwent rehabilitation to be released back to natural habitat or that were transferred to be maintained long-term in captivity, and those that died or were euthanized while under care. Parameters associated with youth and active skeletal growth (absolute lymphocytes, tCa^2+^, ALP) were higher for seals that were eventually released or transferred than those that died or were euthanized. Parameters most likely to be affected by dehydration (BUN, Na^+^, Cl^-^) were higher among animals that died or were euthanized than among those that were released or transferred, as were parameters that may have been increased due to systemic or metabolic disease or exertional rhabdomyolysis due to restraint or exhaustion prior to stranding (AST, ALT, bilirubin, P). CPK was higher among animals that died or were euthanized than those that survived to be released or transferred, but the difference was not statistically significant. CPK activity may not be as sensitive prognostic indicator as AST, ALT, or P.

Strong lymphocyte proliferation has been described in healthy harbor seal newborns reflecting a relative immunocompetence as an evolutionary adaptation to short nursing periods and limited maternal care^8^. This adaptation is proposed to accommodate extended periods of maternal foraging, sometimes lasting between one to fourteen days at a time^9^. Conversely, seals may become transiently lymphocytopenic as a physiologic response to the stress associated with capture and restraint for blood collection. Lymphocytopenia may also result of a failure to mount an adaptive immune response, and therefore a lower likelihood to survive rehabilitation. Lymphocytopenia in grey seal pups presented for rehabilitation has been associated with malnutrition, although hypocalcemia was not^10^. Higher calcium concentrations in Pacific harbor seal pups prior to release following rehabilitation have been attributed to skeletal growth^6^. Therefore, both higher lymphocyte counts and higher tCa^2+^ levels may reflect the young age of animals rather than directly contributing to their clinical outcome, although the role of higher lymphocyte counts in an adaptive immune response cannot be ruled out.

Hematology and blood biochemistry values are influenced by the age, sex, species of the individual, as well as their geographic origin, annual and seasonal variations, and physiologic states such as pregnancy or lactation^11–15^. For example, harp seal pups have been reported to have significantly lower hemoglobin, PCV, and RBC, but higher nucleated RBC counts than pups of other species, which is potentially a consequence of the quicker development of hooded seal pups when compared to harp seal pups^15^. Sex may also influence the results as male hooded seals have been reported to have higher hemoglobin, PCV, and RBC than female harp and hooded seals^15^. Although variations of K^+^ and magnesium (Mg^2+^) were species specific, Ca^2+^, P, Na^+^, Cl^−^, and total CO_2_ were not different across species, but were affected by age^15^. Harp seal pups have been reported to have higher blood P and K^+^ concentrations than other seal species^15^. Season of the year has been shown to influence BUN, Na^+^, Cl^−^, HCT, and hemoglobin in captive harbor seals^12^. These factors likely confound the use of blood parameters alone as prognostic indicators for phocid rehabilitation. Future prospective studies are warranted to account for the potential effects of these factors.

Interestingly, absolute and relative eosinophils were highest among animals that were euthanized compared to those that died naturally and those that survived. Although speculative, chronic parasitic or fungal infection should be considered for seals that do not improve during initial rehabilitation. Although not routinely prescribed at the center, anthelmintic and antifungal treatment may improve the prognoses of animals exhibiting eosinophilia on initial hematology. Erythrocyte concentrations were found to be statistically significantly lower among animals that were euthanized, however the clinical significance of this difference is not clear.

### Predictors of successful release

Changes in several hematologic and plasma biochemical parameters are associated with increased odds of successful release. The univariate model showed that the odds of release decrease with increasing bilirubin, creatinine, P, Na^+^, and Cl^−^. In both univariate and multivariate models, odds of release increase with increasing tCa^2+^ concentrations, and decrease with increasing BUN concentrations. These findings are consistent with the results of the comparison of means presented in Table 2.

Higher tCa^2+^ concentrations are consistent with animals in good planes of nutrition, especially actively growing juveniles, whereas higher bilirubin concentrations may be caused by recent fasting. Other than starvation, physical exertion prior to and during capture and restraint may cause hemolysis and increase total bilirubin. Physical restraint in seals and other species has been associated with temporary hyperkalemia and hyperphosphatemia^10,16,17^. Physical exhaustion immediately prior to stranding and presentation due to the stranding event may cause exertional rhabdomyolysis or predispose the animals to capture myopathy during restraint for blood collection and treatment. Firm physical restraint has been associated with capture myopathy in rehabilitated phocids who are rehydrated enterally via gavage, making it imperative to limit the time and force required to restrain animals^17^. Alternatively, non-pathologic “physiologic jaundice” has been described in healthy neonatal harbor seals, and is a plausible explanation for the observed hyperbilirubinemia when considering a 95.8% juvenile sample population^18^.

Pre-renal azotemia and elevated electrolyte concentrations due to hemoconcentration are not surprising given that stranded pinnipeds are often dehydrated^16,17,19,20^. Increasing BUN, creatinine, Na^+^, and Cl^−^ concentrations were each associated with decreasing odds of release. This suggests that the more severely dehydrated a seal is at presentation, the less likely it is to be ultimately released and highlights the importance of appropriate fluid therapy during the course of rehabilitation. Plasma electrolytes, BUN, and creatinine levels may provide clinical benchmarks to monitor fluid resuscitation.

To date, there are a limited number of studies investigating prognostic indicators of phocids in relation to hematology and/or biochemistry. One previous study of stranded harbor seals in which lower platelet counts and protein concentrations were associated with decreased odds of survival^21^. That study, however, had a smaller sample size, including 64 harbor seal pups at time of admission. Our dataset did not identify similar findings.

As the length of hospitalization increased, so did the odds of successful release. Animals that show initial improvement are likely to be kept hospitalized even if they require a prolonged convalescence. Although evaluation of causes of stranding was beyond the scope of this study, review of the center’s records indicates some seals were thin on presentation and others with soft tissue wounds consistent with shark bites. Most seals were presented in the winter. Time in rehabilitation has been associated with the improvement of the nutritional status of seals over time as reflected by increased triglyceride concentrations^22^. This may contribute to the improved odds of release over time. Seals in poor body condition may be kept under care until they have developed appropriate fat stores prior to release or until milder weather of late winter. The center allows soft tissue wounds to heal by second intention requiring longer periods of hospitalization for those animals that survive until the wound fully heal.

### Predictors of hospitalization

The univariate model showed the number of days in hospital increased as initial amylase concentrations increased and as initial TP, albumin, ALT, P, HCT, and eosinophil concentrations decreased. In both univariate and multivariate models, increasing initial leukocyte concentrations and decreasing initial creatinine, Na^+^, and K^+^ concentrations were associated with longer periods of hospitalization.

Monocytosis and neutrophilia have been reported in marine mammals presented for rehabilitation following severe trauma, inflammation, and infection^6,10,23^. These conditions may require prolonged treatment or convalescence compared to parasitism, a likely cause of eosinophilia, that can be more quickly resolved with empirical anthelmintic therapy. Indeed, parasitism, especially by the lungworm *Parafilaroides decorus*, is common among free-ranging phocids^20,24^.

Increases in amylase concentrations had increased odds of longer duration of hospitalization. Although statistically significant, this analyte alone is unlikely to be clinically significant or to aid prognostication due the small changes in probability per incremental increase.

Increasing concentrations of TP, albumin, Na^+^, P, K^+^, creatinine, and HCT were associated with fewer days of hospitalization. All of these may increase concurrently due to dehydration and could be treated relatively rapidly with fluid therapy begun at or near the time of admission.

Grey seals were more likely to spend longer time in hospital whereas the opposite was true of harp seals. These could be related to differences in the causes of stranding for each species or due to sampling bias. Further research into the proximate causes of stranding for various phocid species in this region is warranted.

Other studies have shown that physical variables such as body mass and weight-to-length ratio at time of admission may be more predictive of the outcomes than clinical pathology for seals presented for rehabilitation^21,25^. The potential effect of these physical variables, proximate causes stranding, and morbidities present at time of admission were outside the scope of this investigation.

The retrospective nature of this study introduced several limitations. The stranding center employs full-time stranding technicians and utilizes a body of trained volunteers to respond to cases of marine mammals found on land and apparently unable to return to the surf. These cases are reported by the public to the center by telephone either directly or via a hotline answering service. Technicians respond to calls and animals that are obviously ill or injured and those that do not flee from approach or resist human contact are transported the center for medical care that may include diagnostics (e.g. fecal examination, hematology, plasma biochemistry) and empirical treatment (e.g. antibiotics, enteral fluids, gavage feeding). The time between presentation and blood collection was not uniform, although only cases in which blood was collected within 48 h of presentation were included. Timing of venipuncture and time to centrifugation of the blood sample could vary based on availability of trained staffs, time of day when the animal arrived at the center, and the operating schedule of the reference laboratory. Additionally, in most cases, the medical record did not clearly indicate if blood was collected prior to or following initial treatments. Medical records gave few details of physical exam findings at the time of presentation or venipuncture, or the condition of the animal and degree of restraint required to obtain blood samples. For this reason, these factors were not included in this study although they could affect hematologic and plasma biochemical parameters. Because the center does not employ a full-time veterinarian on site, seals are rarely evaluated by a veterinarian immediately, although always within 48 h of presentation. By the time the veterinarian arrives, initial triage has been performed, treatments administered, and results of initial bloodwork available for review. Therefore, the results of this study closely approximate the conditions under which the veterinarian must make clinical decisions.

## Conclusion

This retrospective study identified several hematologic and plasma biochemical parameters that differed between free-ranging seals presented for rehabilitation that were successfully released to their natural habitat and those that were not. Furthermore, changes in hematologic and plasma biochemical parameters were identified that were associated with increased odds of survival and longer terms of hospitalization. Generally, bloodwork results that were consistent with juvenile animals carried better prognoses, whereas those consistent with dehydration, systemic disease, or exhaustion carried poorer prognoses. Parenteral fluid therapy during initial presentation may improve the prognosis of seals presented for rehabilitation. Hematology and plasma biochemistry within 48 h of presentation may improve triage and direct treatment plans for seals presented for rehabilitation.
